# Supplementary material for: Repurposing CRISPR-Cas13 systems for robust mRNA trans-splicing
Source: Nat Commun. 2024 Mar 14;15:2325. doi: 10.1038/s41467-024-46172-4 (PMC10940283; doi:10.1038/s41467-024-46172-4)
Supplement: Supplementary file 1 — Supplementary Information [file 41467_2024_46172_MOESM1_ESM.pdf]

Supplementary Figures, Tables & Information

Supplementary Fig. 1. Western blot of Cas13 expression.

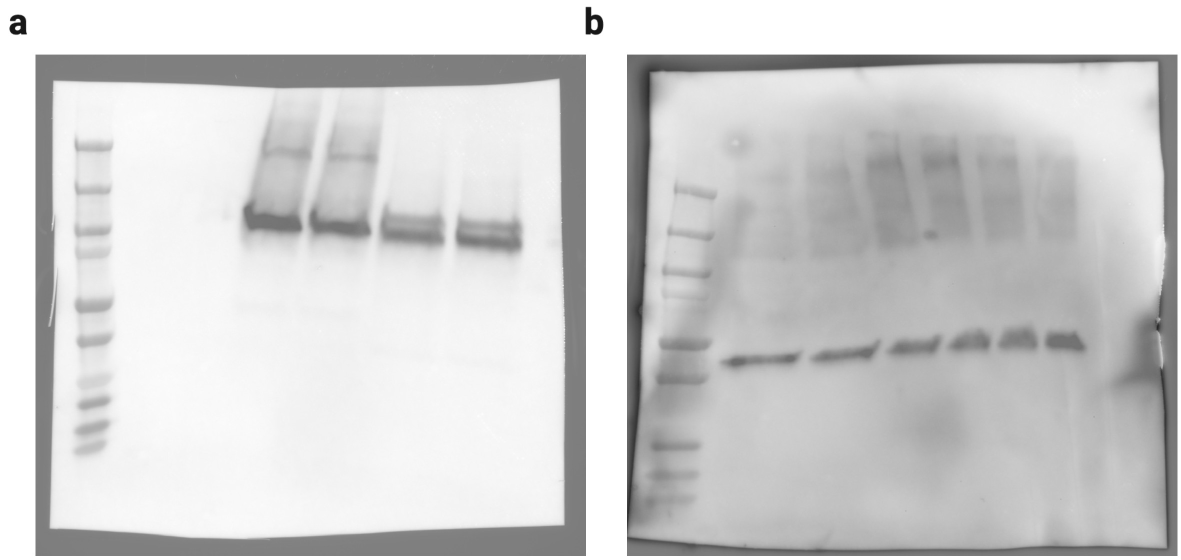

On the left is western blot staining for the HA epitope appended to the c-terminus of the cas13 molecule. Lanes: ladder (1), cells transfected with pUC19 (2,3), cells transfected with Psp-dCas13b (lanes 4,5 | expected protein size is 118kDa) and cells transfected with Rfx-dCas13d (lanes 6,7 | expected protein size is 115kDa). On the right is anti-beta actin staining for as loading control (lanes are the same as left | expected protein size is 42kDa)

Supplementary Fig. 2: Representative gating strategy for flow cytometry experiments.

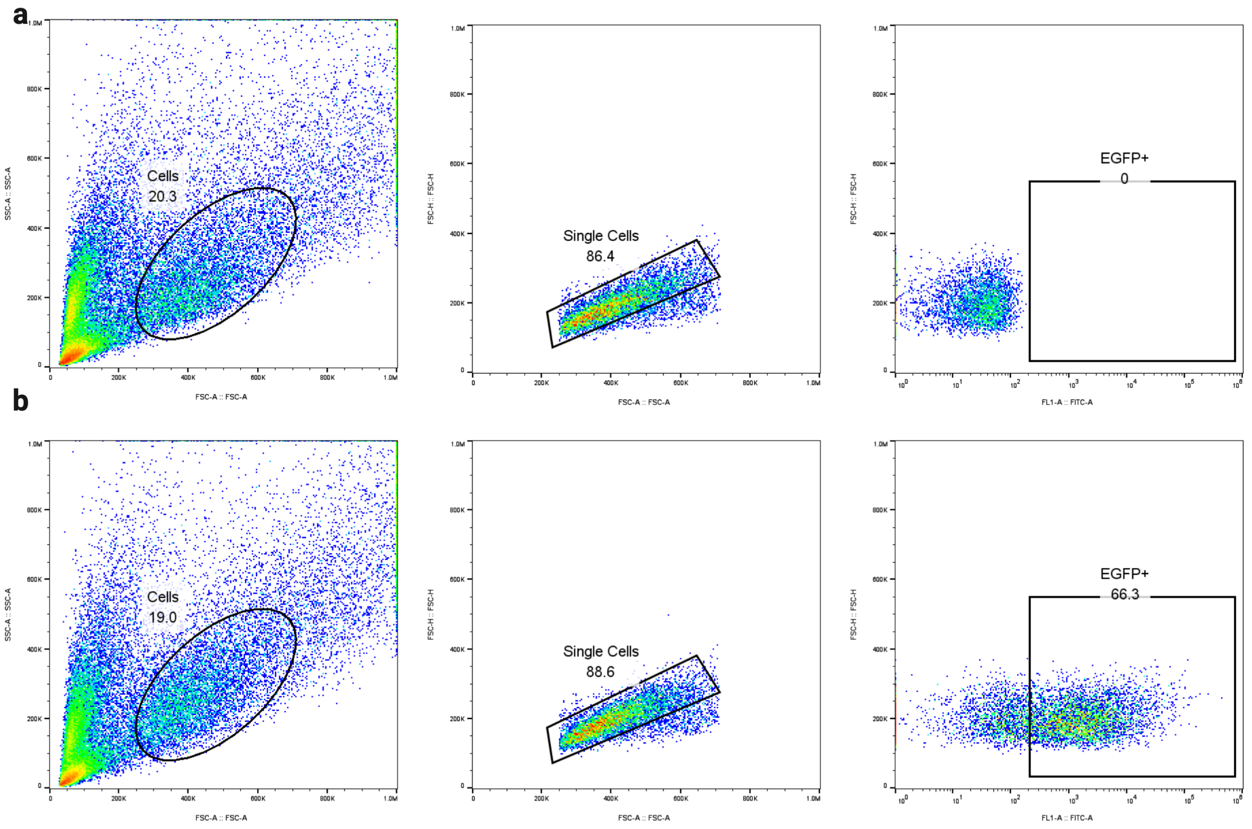

Representative gating strategy for cells that were transfected with the splitGFP reporter alone (a) and with the CRAFT constructs (b). Cell populations were identified by comparing forward and side scatter (left) and passed to discriminate single cells from doublets by comparing forward scatter area to forward scatter height (center). Singlets were then analyzed for expression of EGFP as measured by a 488nm laser (right).

Supplementary Fig.3 CRAFT exhibits robust editing across commonly used immortalized cell lines.

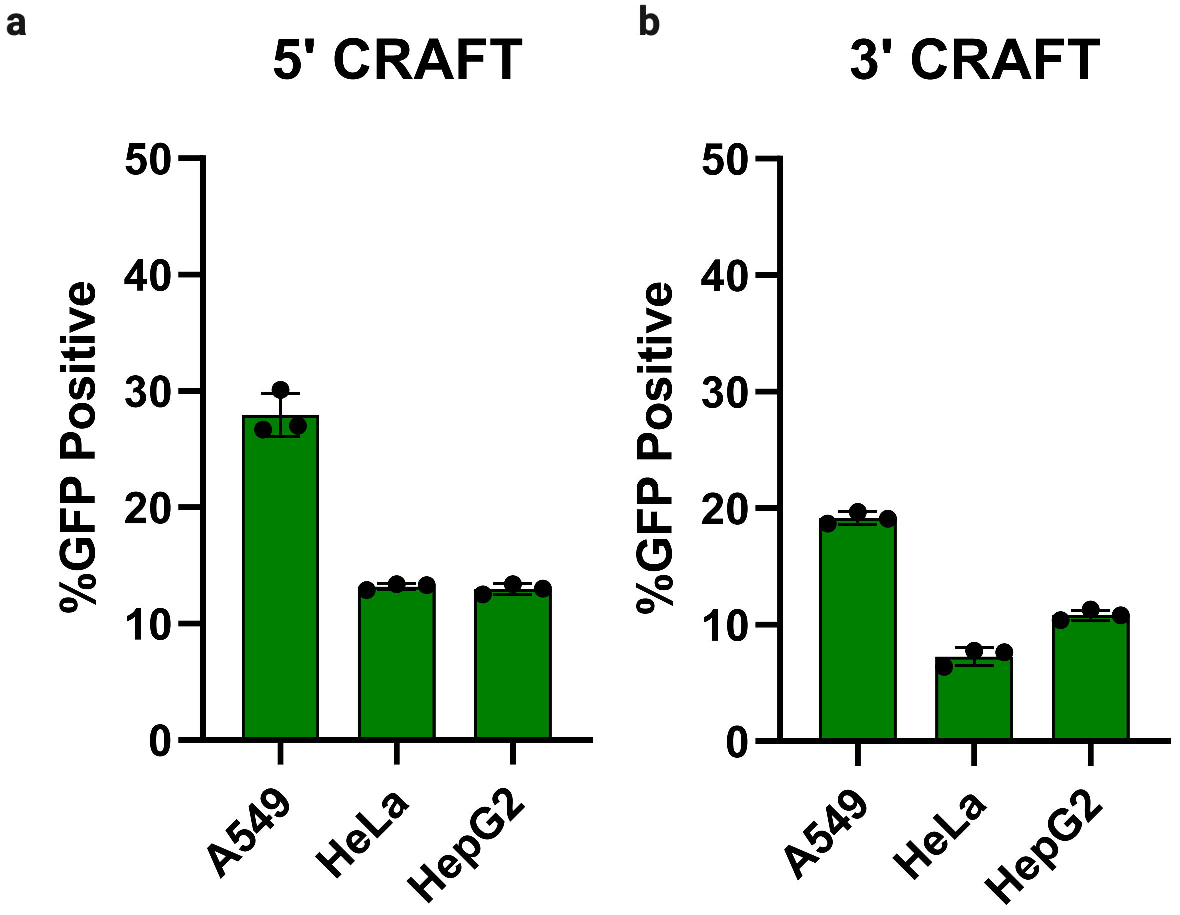

a, Quantitation of flow cytometry for 5’CRAFT in A549 cells, HeLa cells, and HepG2 cells plotted as percent GFP positive cells (*n =3* individual samples, **** means $p < 0.0001$, one-way Anova | data in Source Data file). b, Quantitation of flow cytometry for 3’CRAFT in A549 cells, HeLa cells, and HepG2 cells plotted as percent GFP positive cells (*n =3* individual samples, **** means $p < 0.0001$, one-way Anova | data in Source Data file).

Supplementary Fig. 4. CRAFT can be used to reprogram proteins by editing the mRNA transcripts which encode them.

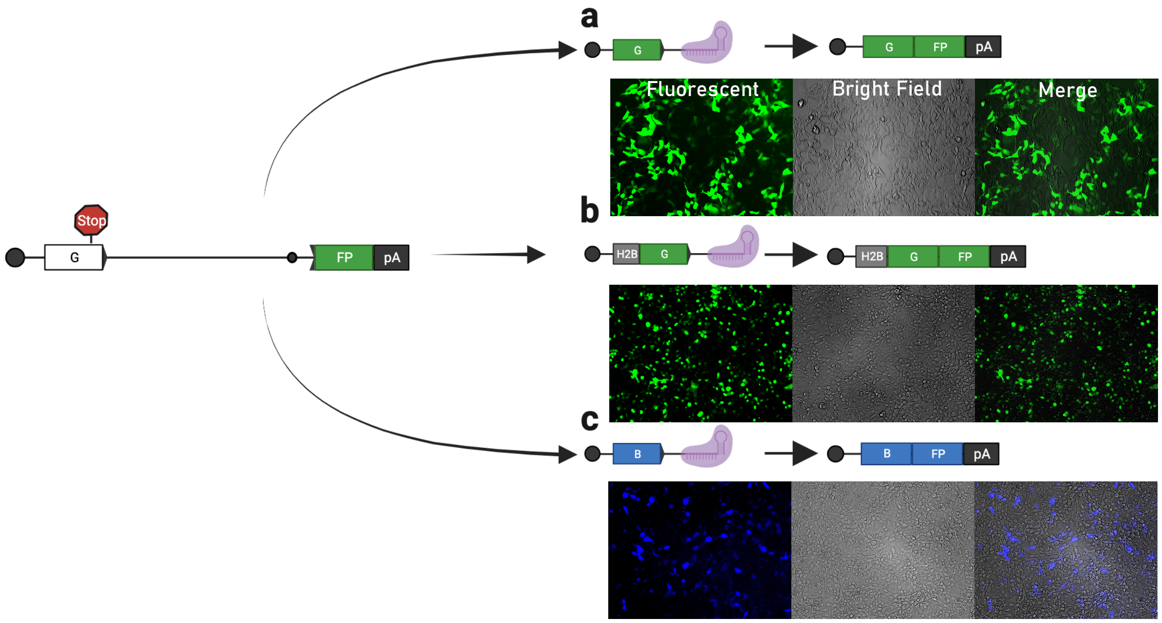

Schematic of 5’CRAFT RNP to restore EGFP expression and resulting mRNA transcript. Representative images of cells transfected with 5’splitGFP reporter, dPspCas13b and 5’ rcRNA to restore EGFP expression (a). Schematic of 5’CRAFT RNP to restore EGFP expression and localize EGFP expression to the nucleus along with the resulting mRNA transcript. Representative images of cells transfected with 5’splitGFP reporter, dPspCas13b and 5’ rcRNA (b). Schematic of 5’CRAFT RNP to convert EGFP>BFP and resulting mRNA transcript. Representative images of cells transfected with 5’splitGFP reporter, dPspCas13b and 5’ rcRNA to convert EGFP to BFP (c).

Supplementary Fig. 5. rcRNA targeting the *LMNA* intron 10/11 of splitGFP reporter generate chimeric mRNA fusions by splicing into endogenous *LMNA* pre-mRNA.

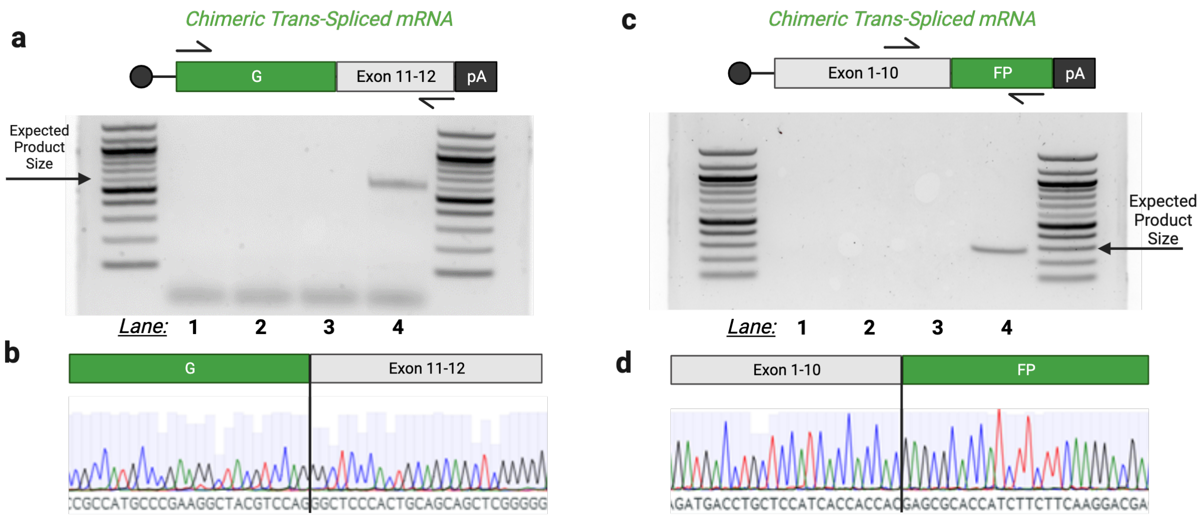

a, Schematic of chimeric RNA made by 5' CRAFT rcRNA containing the first half of the EGFP coding region splicing into the endogenous *LMNA* transcript, primers used to amplify this chimeric product RNA collected from cells are depicted by half arrows on the schematic (top). Gel of PCR products amplifying the chimeric trans-spliced RNA (expected product size = 609bp) dPspCas13b (lane 1), 5' rcRNA (lane 2), dPspCas13b and a 5'rcRNA that does not target the *LMNA* intron (lane 3), or dPspCas13b and a 5'rcRNA that does target the *LMNA* intron (lane 4) (bottom). b, Sanger sequencing trace across the splice junction, for reference the vertical line denotes the junction between the two joined sequences. c, Schematic of chimeric RNA made by 3' CRAFT rcRNA containing the second half of the EGFP coding region splicing into the endogenous *LMNA* transcript, primers used to amplify this chimeric product RNA collected from cells are depicted by half arrows on the schematic (top). Gel of PCR products amplifying the chimeric trans-spliced RNA (expected product size = 301bp) dRfxCas13d (lane 1), 3'

rcRNA (lane 2), dRfxCas13d and a 3'rcRNA that does not target the *LMNA* intron (lane 3), or dRfxCas13d and a 3'rcRNA that does target the *LMNA* intron (lane 4) (bottom). d, Sanger sequencing trace across the splice junction, for reference.

Supplementary Fig. 6: Analysis of on-target editing outcomes with CRISPResso2.

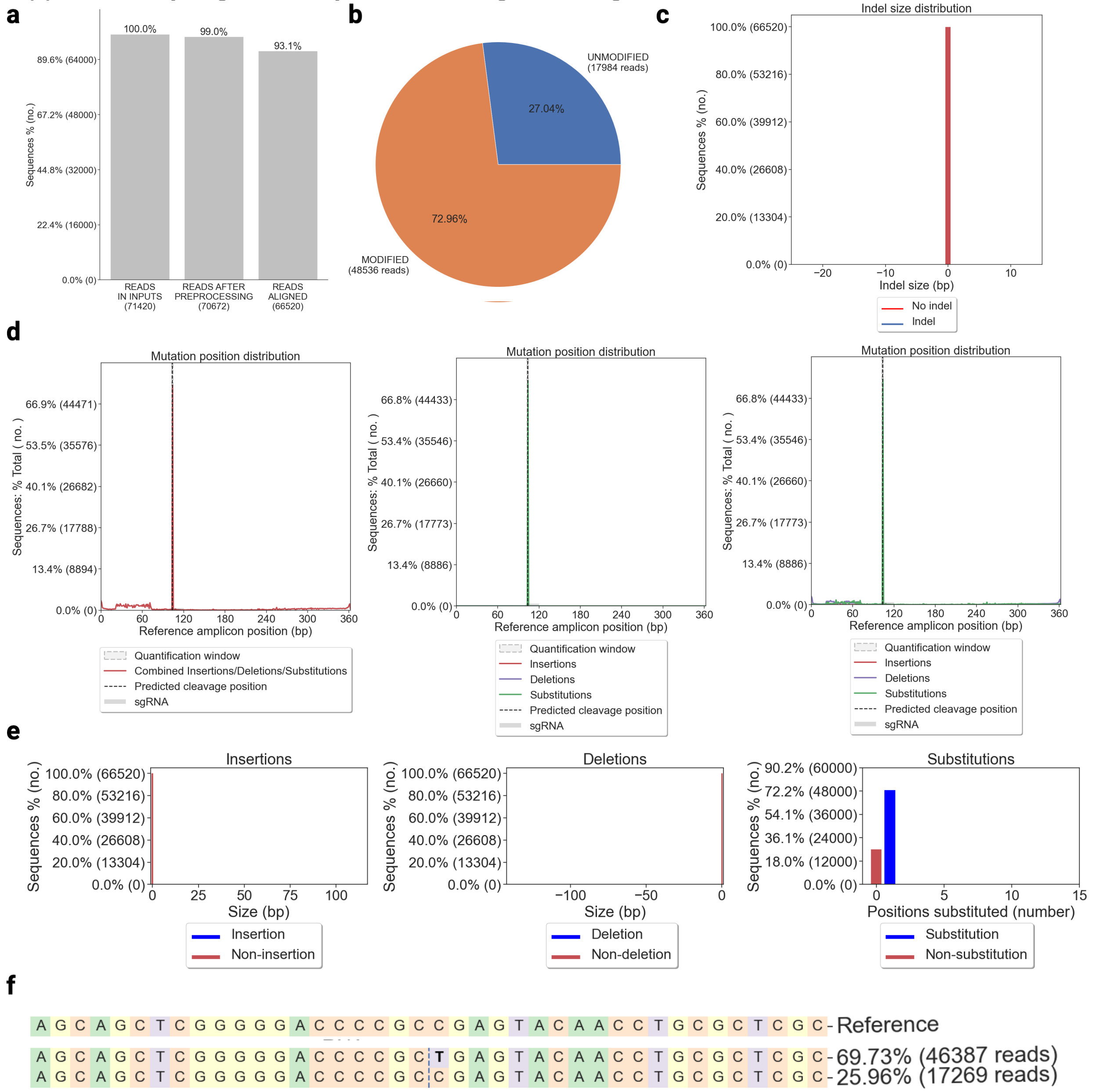

This plot was made by analyzing amplicon sequencing reads from the 3'CRAFT approach to edit the endogenous *LMNA* transcript. a, Statistics on processing of input fastq file following quality check, read trimming and alignment to reference trans-spliced RNA. b, Pie chart indicating the percent of modified reads from reference transcipt. c, Plot of indel frequency and size in amplicons relative to reference. d, Plot of mutation frequency as a function position along reference. e, Plot of mutation frequency as a function of the

mutation size for each type of mutation. f, Majority RNA species of either wild type or only containing the SNP of interest. Trans-splicing is measured using the following formula:

$$\%\text{Editing} = 100 * \frac{\%\text{reads C}}{\%\text{reads C} + \%\text{reads T}}$$

The example shown would be calculated as such:

$$\%\text{Editing} = 100 * \frac{25.96}{25.96 + 69.73} = 27.13\%$$

Supplementary Fig. 7: CRAFT reduces Progerin mRNA in Hutchinson-Gilford Progeria Syndrome patient fibroblasts.

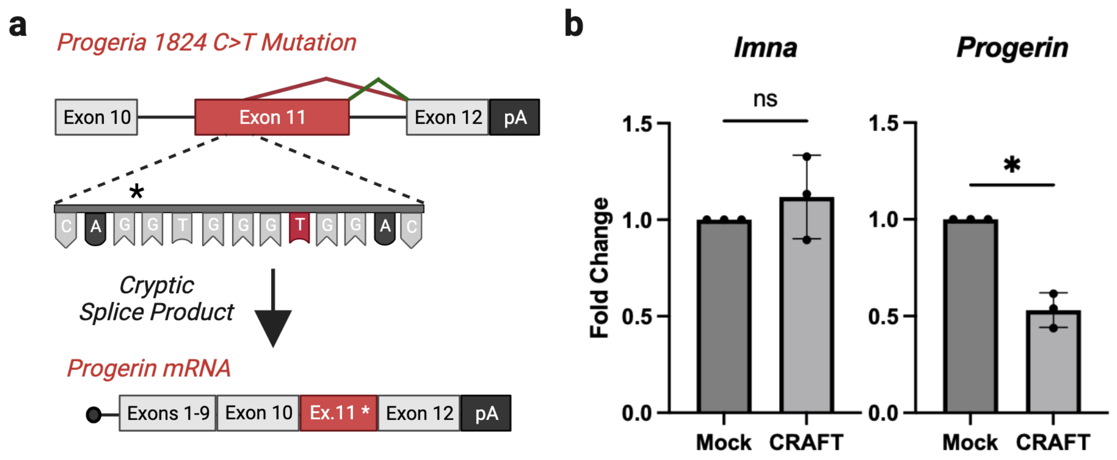

a, schematic of progeria mutation and cyptic splicing pattern show by the red lines compared to normal splicing shown in green. b, Fold change in *LMNA* and *progerin* transcript abundance as determined by qPCR through ddCt (*n =3* individual samples, * p = 0.0118, paired two-tailed t-test).

Supplementary Fig. 8: Comparison of CRAFT and Splice Editing platforms.

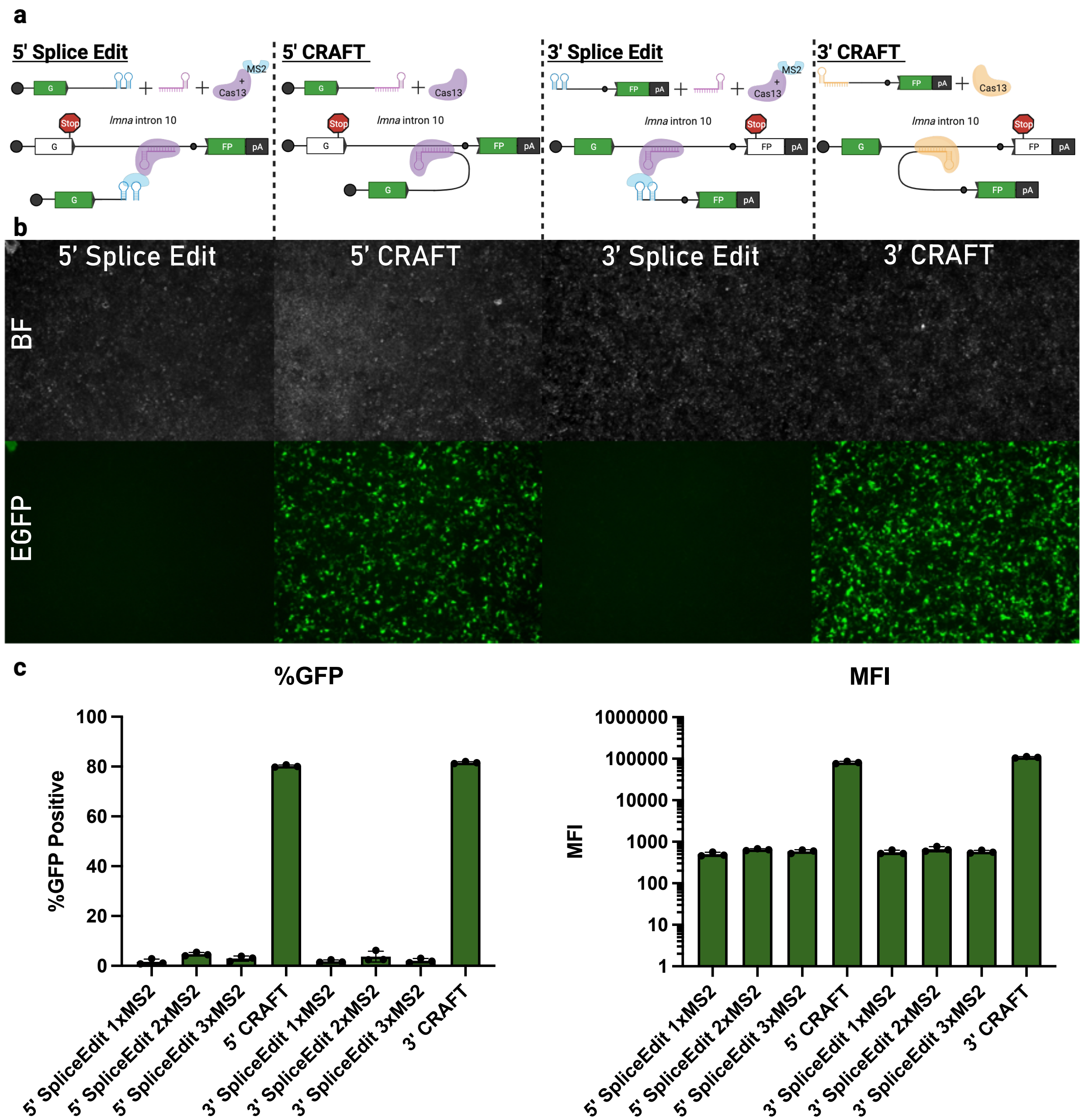

a, Schematic Splice Edit and CRAFT constructs. b, Representative fluorescent images of cells transfected with *LMNA* SplitGFP reporter plasmid containing premature stop codon and either Splice Edit or CRAFT machinery. c, Quantification by flow cytometry presented

as percent GFP positive cells and MFI (right) (*n =3* individual samples | data in Source Data file)

Supplementary Fig. 9: *in vivo* evaluation of CRAFT in the *mdx* mouse.

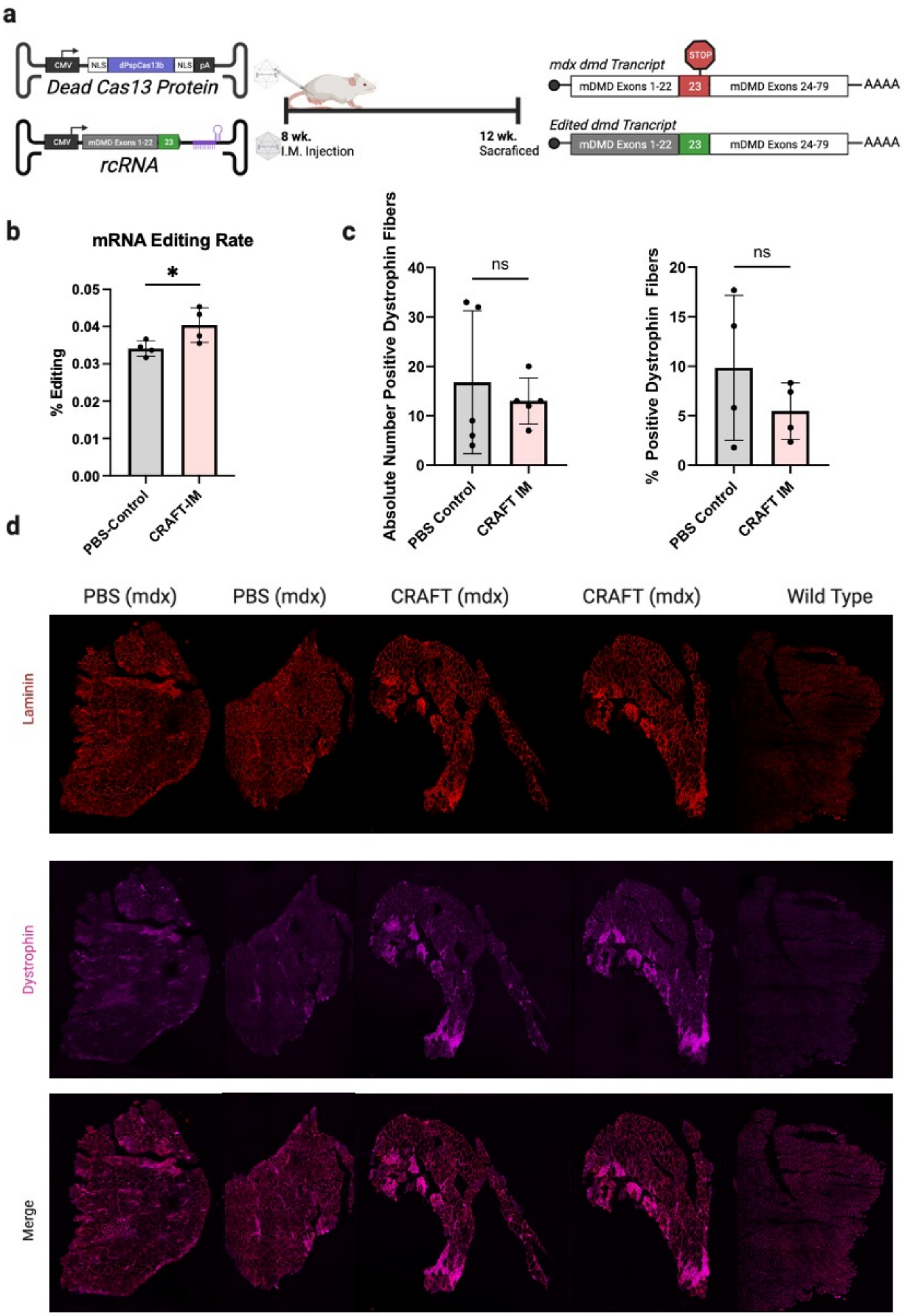

a, schematic of AAV cassettes (left), experiment timeline (center), and RNA editing outcomes (right). b, quantification of mRNA trans-splicing by targeted amplicon sequencing (*n* = 4 mice per cohort, *p = 0.049, unpaired two-tailed t-test). c, quantification of dystrophin positive fibers. d, representative confocal images of the TA of injected mice.

Supplementary Table 1: Sequences of Essential Constructs

rcRNA Cassettes

CMV Promoter | Direct Repeat | Esp3i Cloning Sites | Spacer Sequence | Hemi-Intron | cDNA | SV40 Polyadenylation Sequence | *Multiple Cloning Site*

5' rcRNA with Esp3i Cutsites:

AGTAATCAATTACGGGGTCATTAGTTCATAGCCCATATATGGAGTTCCGCGTTACAT
AACTTACGGTAAATGGCCCGCCTGGCTGACCGCCCAACGACCCCCGCCCATTGAC
GTCAATAATGACGTATGTTCCCATAGTAACGCCAATAGGGACTTTCCATTGACGTC
AATGGGTGGAGTATTTACGGTAAACTGCCCACTTGGCAGTACATCAAGTGTATCAT
ATGCCAAGTACGCCCCCTATTGACGTCAATGACGGTAAATGGCCCGCCTGGCATT
ATGCCCAGTACATGACCTTATGGGACTTTCCTACTTGGCAGTACATCTACGTATTAG
TCATCGCTATTACCATGCTGATGCGGTTTTGGCAGTACATCAATGGGCGTGGATAG
CGGTTTGACTCACGGGGATTTCCAAGTCTCCACCCCATTGACGTCAATGGGAGTTT
GTTTTGGCACCAAAATCAACGGGACTTTCCAAAATGTCGTAACAACTCCGCCCCAT
TGACGCAAATGGGCGGTAGGCGTGTACGGTGGGAGGTCTATATAAGCAGAGCTG
GTTTAGTGAACCGTCAGAT*CAGATCTTTGTCGATCCTACCATCCACTCGACACACC*
*CGCCAGCGGCCGCGCCAAC*ATGgtgagcaagggcgaggagctgttcaccggggtggtgcccatcctggt
cgagctggacggcgacgtaaacggccacaagttcagcgtgtccggcgagggcgagggcgatgccacctacggcaag
ctgaccctgaagttcatctgcaccaccggcaagctgcccgtgccctggcccaccctcgtgaccaccctgacctacggcgt
gcagtgcttcagccgctaccccgaccacatgaagcagcacgacttcttcaagtccgccatgcccgaaggctacgtccag
GTAAGAGAGCTCGTTGCGATATTATcgagacgTCTATCATGCAGTCAGcgtctcCGTTGT
GGAAGGTCCAGTTTTGAGGGGCTATTACAAC*GATCCGCAGGCCTCTGCTAGCTTG*

*ACTGACTGAGATACAGCGTACCTT*CAGCTCACAGACATGATAAGATACATTGATGA
GTTTGGACAAACCACAACTAGAATGCAGTGAAAAAAATGCTTTATTTGTGAAATTTG
TGATGCTATTGCTTTATTTGTAACCATTATAAGCTGCAATAAACAAGTTAACAACAAC
AATTGCATTCATTTTATGTTTCAGGTTCAGGGGGAGGTGTGGGAGGTTTTTTAAAG
CAAGTAAAACCTCTACAAATGTGGTATTGG

5’ rcRNA with best guide:

AGTAATCAATTACGGGGTCATTAGTTCATAGCCCATATATGGAGTTCCGCGTTACAT
AACTTACGGTAAATGGCCCGCCTGGCTGACCGCCCAACGACCCCCGCCCATTGAC
GTCAATAATGACGTATGTTCCCATAGTAACGCCAATAGGGACTTTCCATTGACGTC
AATGGGTGGAGTATTTACGGTAAACTGCCCACTTGGCAGTACATCAAGTGTATCAT
ATGCCAAGTACGCCCCCTATTGACGTCAATGACGGTAAATGGCCCGCCTGGCATT
ATGCCCAGTACATGACCTTATGGGACTTTCCTACTTGGCAGTACATCTACGTATTAG
TCATCGCTATTACCATGCTGATGCGGTTTTGGCAGTACATCAATGGGCGTGGATAG
CGGTTTGACTCACGGGGATTTCCAAGTCTCCACCCCATTGACGTCAATGGGAGTTT
GTTTTGGCACCAAAATCAACGGGACTTTCCAAAATGTCGTAACAACTCCGCCCCAT
TGACGCAAATGGGCGGTAGGCGTGTACGGTGGGAGGTCTATATAAGCAGAGCTG
GTTTAGTGAACCGTCAGAT*CAGATCTTTGTCGATCCTACCATCCACTCGACACACC*
*CGCCAGCGGCCGCGCCAAC*ATGgtgagcaagggcgaggagctgttcaccggggtggtgcccatcctggt
cgagctggacggcgacgtaaacggccacaagttcagcgtgtccggcgagggcgagggcgatgccacctacggcaag
ctgaccctgaagttcatctgcaccaccggcaagctgcccgtgccctggcccaccctcgtgaccaccctgacctacggcgt
gcagtgcttcagccgctaccccgaccacatgaagcagcacgacttcttcaagtccgccatgcccgaaggctacgtccag
GTAAGAGAGCTCGTTGCGATATTATTTCTAGCTACTCTGAGCTTAAGAGGAAAAGG

TTGTGGAAGGTCCAGTTTTGAGGGGCTATTACAAC*GATCCGCAGGCCTCTGCTAG CTTGACTGACTGAGATACAGCGTACCTT*CAGCTCACAGACATGATAAGATACATTG ATGAGTTTGGACAAACCACAACTAGAATGCAGTGAAAAAAATGCTTTATTTGTGAAA TTTGTGATGCTATTGCTTTATTTGTAACCATTATAAGCTGCAATAAACAAGTTAACAA CAACAATTGCATTCATTTTATGTTTCAGGTTCAGGGGGAGGTGTGGGAGGTTTTTTA AAGCAAGTAAAACCTCTACAAATGTGGTATTGG

3' rcRNA with Esp3i cutsites:

TAGTAATCAATTACGGGGTCATTAGTTCATAGCCCATATATGGAGTTCCGCGTTACA TAACTTACGGTAAATGGCCCGCCTGGCTGACCGCCCAACGACCCCCGCCCATTGA CGTCAATAATGACGTATGTTCCCATAGTAACGCCAATAGGGACTTTCCATTGACGT CAATGGGTGGAGTATTTACGGTAAACTGCCCACTTGGCAGTACATCAAGTGTATCA TATGCCAAGTACGCCCCCTATTGACGTCAATGACGGTAAATGGCCCGCCTGGCAT TATGCCCAGTACATGACCTTATGGGACTTTCCTACTTGGCAGTACATCTACGTATTA GTCATCGCTATTACCATGCTGATGCGGTTTTGGCAGTACATCAATGGGCGTGGATA GCGGTTTGACTCACGGGGATTTCCAAGTCTCCACCCCATTGACGTCAATGGGAGTT TGTTTTGGCACCAAAATCAACGGGACTTTCCAAAATGTCGTAACAACTCCGCCCCA TTGACGCAAATGGGCGGTAGGCGTGTACGGTGGGAGGTCTATATAAGCAGAGCTG GTTTAGTGAACCGTCAGATC*AGATCTTTGTCGATCCTACCATCCACTCGACACACC CGCCAGCGGCCGCG*AACCCCTACCAACTGGTCGGGGTTTGAAACcgagacgTCTAT CATGCAGTCAGcgtctcCCCGCGGAACATTATTATAACGATTGCTCGGGCTGAGGGA AGGACTGTCCTGGGGACTGGAATACTAACTGATATCTCTTCTTTTTCTTTTTCCCCA AAACAGGAGCGCACCATCTTCTTCAAGGACGACGGCAACTACAAGACCCGCGCCG

AGGTGAAGTTCGAGGGCGACACCCTGGTGAACCGCATCGAGCTGAAGGGCATCG
ACTTCAAGGAGGACGGCAACATCCTGGGGCACAAGCTGGAGTACAACTACAACAG
CCACAACGTCTATATCATGGCCGACAAGCAGAAGAACGGCATCAAGGTGAACTTC
AAGATCCGCCACAACATCGAGGACGGCAGCGTGCAGCTCGCCGACCACTACCAG
CAGAACACCCCCATCGGCGACGGCCCCGTGCTGCTGCCCGACAACCACTACCTG
AGCACCCAGTCCGCCCTGAGCAAAGACCCCAACGAGAAGCGCGATCACATGGTC
CTGCTGGAGTTCGTGACCGCCGCCGGGATCACTCTCGGCATGGACGAGCTGTACA
AGTAA*GGATCCGCAGGCCTCTGCTAGCTTGACTGACTGAGATACAGCGTACCTT*C
AGCTCACAGACATGATAAGATACATTGATGAGTTTGGACAAACCACAACTAGAATG
CAGTGAAAAAAATGCTTTATTTGTGAAATTTGTGATGCTATTGCTTTATTTGTAACCA
TTATAAGCTGCAATAAACAAGTTAACAACAACAATTGCATTCATTTTATGTTTCAGGT
TCAGGGGGAGGTGTGGGAGGTTTTTTAAAGCAAGTAAAACCTCTACAAATGTGGTA
TTGG

3’ rcrna with best guide:

TAGTAATCAATTACGGGGTCATTAGTTCATAGCCCATATATGGAGTTCCGCGTTACA
TAACTTACGGTAAATGGCCCGCCTGGCTGACCGCCCAACGACCCCCGCCCATTGA
CGTCAATAATGACGTATGTTCCCATAGTAACGCCAATAGGGACTTTCCATTGACGT
CAATGGGTGGAGTATTTACGGTAAACTGCCCACTTGGCAGTACATCAAGTGTATCA
TATGCCAAGTACGCCCCCTATTGACGTCAATGACGGTAAATGGCCCGCCTGGCAT
TATGCCCAGTACATGACCTTATGGGACTTTCCTACTTGGCAGTACATCTACGTATTA
GTCATCGCTATTACCATGCTGATGCGGTTTTGGCAGTACATCAATGGGCGTGGATA
GCGGTTTGACTCACGGGGATTTCCAAGTCTCCACCCCATTGACGTCAATGGGAGTT

TGTTTTGGCACCAAAATCAACGGGACTTTCCAAAATGTCGTAACAACTCCGCCCCA

TTGACGCAAATGGGCGGTAGGCGTGTACGGTGGGAGGTCTATATAAGCAGAGCTG

GTTTAGTGAACCGTCAGATC*AGATCTTTGTCGATCCTACCATCCACTCGACACACC*

*CGCCAGCGGCCGCG*AACCCCTACCAACTGGTCGGGGTTTGAAACCAAATCCAGAC

CCTTGTCCACTGCTCCCACAGGAATATTCCATGGCATCACCGCGGAACATTATTAT

AACGATTGCTCGGGCTGAGGGAAGGACTGTCCTGGGGACTGGAATACTAACTGAT

ATCTCTTCTTTTTCTTTTTCCCCAAAACAGGAGCGCACCATCTTCTTCAAGGACGAC

GGCAACTACAAGACCCGCGCCGAGGTGAAGTTCGAGGGCGACACCCTGGTGAAC

CGCATCGAGCTGAAGGGCATCGACTTCAAGGAGGACGGCAACATCCTGGGGCAC

AAGCTGGAGTACAACTACAACAGCCACAACGTCTATATCATGGCCGACAAGCAGAA

GAACGGCATCAAGGTGAACTTCAAGATCCGCCACAACATCGAGGACGGCAGCGTG

CAGCTCGCCGACCACTACCAGCAGAACACCCCCATCGGCGACGGCCCCGTGCTG

CTGCCCGACAACCACTACCTGAGCACCCAGTCCGCCCTGAGCAAAGACCCCAACG

AGAAGCGCGATCACATGGTCCTGCTGGAGTTCGTGACCGCCGCCGGGATCACTCT

CGGCATGGACGAGCTGTACAAGTAA*GGATCCGCAGGCCTCTGCTAGCTTGACTGA*

*CTGAGATACAGCGTACCTT*CAGCTCACAGACATGATAAGATACATTGATGAGTTTG

GACAAACCACAACTAGAATGCAGTGAAAAAAATGCTTTATTTGTGAAATTTGTGATG

CTATTGCTTTATTTGTAACCATTATAAGCTGCAATAAACAAGTTAACAACAACAATTG

CATTCATTTTATGTTTCAGGTTCAGGGGGAGGTGTGGGAGGTTTTTTAAAGCAAGT

AAAACCTCTACAAATGTGGTATTGG

CMV Promoter | Esp3i Cloning Sites | SMaRT Spacer Sequence | Hemi-Intron | cDNA |

SV40 Polyadenylation Sequence | *Multiple Cloning Site*

5’ SMaRT PTM:

AGTAATCAATTACGGGGTCATTAGTTCATAGCCCATATATGGAGTTCCGCGTTACAT

AACTTACGGTAAATGGCCCGCCTGGCTGACCGCCCAACGACCCCCGCCCATTGAC

GTCAATAATGACGTATGTTCCCATAGTAACGCCAATAGGGACTTTCCATTGACGTC

AATGGGTGGAGTATTTACGGTAAACTGCCCACTTGGCAGTACATCAAGTGTATCAT

ATGCCAAGTACGCCCCCTATTGACGTCAATGACGGTAAATGGCCCGCCTGGCATT

ATGCCCAGTACATGACCTTATGGGACTTTCCTACTTGGCAGTACATCTACGTATTAG

TCATCGCTATTACCATGCTGATGCGGTTTTGGCAGTACATCAATGGGCGTGGATAG

CGGTTTGACTCACGGGGATTTCCAAGTCTCCACCCCATTGACGTCAATGGGAGTTT

GTTTTGGCACCAAAATCAACGGGACTTTCCAAAATGTCGTAACAACTCCGCCCCAT

TGACGCAAATGGGCGGTAGGCGTGTACGGTGGGAGGTCTATATAAGCAGAGCTG

GTTTAGTGAACCGTCAGAT*CAGATCTTTGTCGATCCTACCATCCACTCGACACACC*

*CGCCAGCGGCCGCGCCAAC*ATGgtgagcaagggcgaggagctgttcaccggggtggtgcccatcctggt

cgagctggacggcgacgtaaacggccacaagttcagcgtgtccggcgagggcgagggcgatgccacctacggcaag

ctgaccctgaagttcatctgcaccaccggcaagctgcccgtgccctggcccaccctcgtgaccaccctgacctacggcgt

gcagtgcttcagccgctaccccgaccacatgaagcagcacgacttcttcaagtccgccatgcccgaaggctacgtccag

GTAAGAGAGCTCGTTGCGATATTATcgagacgTCTATCATGCAGTCAGcgtctcC*GATCC*

*GCAGGCCTCTGCTAGCTTGACTGACTGAGATACAGCGTACCTT*CAGCTCACAGAC

ATGATAAGATACATTGATGAGTTTGGACAAACCACAACTAGAATGCAGTGAAAAAA

TGCTTTATTTGTGAAATTTGTGATGCTATTGCTTTATTTGTAACCATTATAAGCTGCA

ATAAACAAGTTAACAACAACAATTGCATTCATTTTATGTTTCAGGTTCAGGGGGAGG

TGTGGGAGGTTTTTTAAAGCAAGTAAAACCTCTACAAATGTGGTATTGG

3’ SMaRT PTM:

TAGTAATCAATTACGGGGTCATTAGTTCATAGCCCATATATGGAGTTCCGCGTTACA

TAACTTACGGTAAATGGCCCGCCTGGCTGACCGCCCAACGACCCCCGCCCATTGA

CGTCAATAATGACGTATGTTCCCATAGTAACGCCAATAGGGACTTTCCATTGACGT

CAATGGGTGGAGTATTTACGGTAAACTGCCCACTTGGCAGTACATCAAGTGTATCA

TATGCCAAGTACGCCCCCTATTGACGTCAATGACGGTAAATGGCCCGCCTGGCAT

TATGCCCAGTACATGACCTTATGGGACTTTCCTACTTGGCAGTACATCTACGTATTA

GTCATCGCTATTACCATGCTGATGCGGTTTTGGCAGTACATCAATGGGCGTGGATA

GCGGTTTGACTCACGGGGATTTCCAAGTCTCCACCCCATTGACGTCAATGGGAGTT

TGTTTTGGCACCAAAATCAACGGGACTTTCCAAAATGTCGTAACAACTCCGCCCCA

TTGACGCAAATGGGCGGTAGGCGTGTACGGTGGGAGGTCTATATAAGCAGAGCTG

GTTTAGTGAACCGTCAGATC*AGATCTTTGTCGATCCTACCATCCACTCGACACACC*

*CGCCAGCGGCCGCG*cgagacgTCTATCATGCAGTCAGcgtctcCCCGCGGAACATTATT

ATAACGATTGCTCGGGCTGAGGGAAGGACTGTCCTGGGGACTGGAATACTAACTG

ATATCTCTTCTTTTTCTTTTTCCCCAAAACAGGAGCGCACCATCTTCTTCAAGGACG

ACGGCAACTACAAGACCCGCGCCGAGGTGAAGTTCGAGGGCGACACCCTGGTGA

ACCGCATCGAGCTGAAGGGCATCGACTTCAAGGAGGACGGCAACATCCTGGGGC

ACAAGCTGGAGTACAACTACAACAGCCACAACGTCTATATCATGGCCGACAAGCA

GAAGAACGGCATCAAGGTGAACTTCAAGATCCGCCACAACATCGAGGACGGCAGC

GTGCAGCTCGCCGACCACTACCAGCAGAACACCCCCATCGGCGACGGCCCCGTG

CTGCTGCCCGACAACCACTACCTGAGCACCCAGTCCGCCCTGAGCAAAGACCCCA

ACGAGAAGCGCGATCACATGGTCCTGCTGGAGTTCGTGACCGCCGCCGGGATCA

CTCTCGGCATGGACGAGCTGTACAAGTAA*GGATCCGCAGGCCTCTGCTAGCTTGA*

*CTGACTGAGATACAGCGTACCTT*CAGCTCACAGACATGATAAGATACATTGATGAG

TTTGGACAAACCACAACTAGAATGCAGTGAAAAAAATGCTTTATTTGTGAAATTTGT

GATGCTATTGCTTTATTTGTAACCATTATAAGCTGCAATAAACAAGTTAACAACAAC

AATTGCATTCATTTTATGTTTCAGGTTCAGGGGGAGGTGTGGGAGGTTTTTTAAAG

CAAGTAAAACCTCTACAAATGTGGTATTGG

SplitGFP Reporters

CBh Promoter | Intron | NheI Cut Site | cDNA | SV40 Polyadenylation Sequence | *Multiple Cloning Site*

Split GFP NheI:

Tacataacttacggtaaatggcccgcctggctgaccgcccaacgacccccgcccattgacgtcaataatgacgtatgttc

ccatagtaacgccaatagggactttccattgacgtcaatgggtggagtatttacggtaaactgcccacttggcagtacatca

agtgtatcatatgccaagtacgccccctattgacgtcaatgacggtaaatggcccgcctggcattatgcccagtacatgacc

ttatgggactttcctacttggcagtacatctacgtattagtcatcgctattaccatggtcgaggtgagccccacgttctgcttcac

tctccccatctcccccccctccccacccccaattttgtatttatttattttttaattattttgtgcagcgatgggggcggggggggg

gggggggcgcgcgccaggcggggcggggcggggcgaggggcggggcggggcgaggcggagaggtgcggcggc

agccaatcagagcggcgcgctccgaaagtttccttttatggcgaggcggcggcggcggcggccctataaaaagcgaag

cgcgcggcgggcgggagtcgctgcgacgctgccttcgccccgtgccccgctccgccgccgcctcgcgccgcccgcccc

ggctctgactgaccgcgttactcccacaggtgagcgggcgggacggcccttctcctccgggctgtaattagctgagcaag

aggtaagggtttaagggatggttggttggtggggtattaatgtttaattacctggagcacctgcctgaaatcactttttttcaggtt

gg*Accggtcgccacc*ATGgtgagcaagggcgaggagctgttcaccggggtggtgcccatcctggtcgagctggacg
gcgacgtaaacggccacaagttcagcgtgtccggcgagggcgagggcgatgccacctacggcaagctgaccctgaa
gttcatctgcaccaccggcaagctgcccgtgccctggcccaccctcgtgaccaccctgacctacggcgtgcagtgcttcag
ccgctaccccgaccacatgaagcagcacgacttcttcaagtccgccatgcccgaaggctacgtccagGCTAGCag
GAGCGCACCATCTTCTTCAAGGACGACGGCAACTACAAGACCCGCGCCGAGGTGA
AGTTCGAGGGCGACACCCTGGTGAACCGCATCGAGCTGAAGGGCATCGACTTCAA
GGAGGACGGCAACATCCTGGGGCACAAGCTGGAGTACAACTACAACAGCCACAAC
GTCTATATCATGGCCGACAAGCAGAAGAACGGCATCAAGGTGAACTTCAAGATCC
GCCACAACATCGAGGACGGCAGCGTGCAGCTCGCCGACCACTACCAGCAGAACA
CCCCCATCGGCGACGGCCCCGTGCTGCTGCCCGACAACCACTACCTGAGCACCC
AGTCCGCCCTGAGCAAAGACCCCAACGAGAAGCGCGATCACATGGTCCTGCTGGA
GTTCGTGACCGCCGCCGGGATCACTCTCGGCATGGACGAGCTGTACAAGTAA*CAT*
*ATGTCT*TGATCATAATCAGCCATACCACATTTGTAGAGGTTTTACTTGCTTTAAAAAA
CCTCCCACACCTCCCCCTGAACCTGAAACATAAAATGAATGCAATTGTTGTTGTTAA
CTTGTTTATTGCAGCTTATAATGGTTACAAATAAAGCAATAGCATCACAAATTTCACA
AATAAAGCATTTTTTTCACTGCATTCTAGTTGTGGTTTGTCCAAACTCATCAATGTAT
CTTA

SplitGFP LMNA intron 10/11:

Tacataacttacggtaaatggcccgcctggctgaccgcccaacgacccccgcccattgacgtcaataatgacgtatgttc
ccatagtaacgccaatagggactttccattgacgtcaatgggtggagtatttacggtaaactgcccacttggcagtacatca
agtgtatcatatgccaagtacgccccctattgacgtcaatgacggtaaatggcccgcctggcattatgcccagtacatgacc
ttatgggactttcctacttggcagtacatctacgtattagtcatcgctattaccatggtcgaggtgagccccacgttctgcttcac

tctccccatctcccccccctccccacccccaattttgtatttatttattttttaattattttgtgcagcgatgggggcggggggggg

gggggggcgcgcgccaggcggggcggggcggggcgaggggcggggcggggcgaggcggagaggtgcggcggc

agccaatcagagcggcgcgctccgaaagtttccttttatggcgaggcggcggcggcggcggccctataaaaagcgaag

cgcgcggcgggcgggagtcgctgcgacgctgccttcgccccgtgccccgctccgccgccgcctcgcgccgcccgcccc

ggctctgactgaccgcgttactcccacaggtgagcgggcgggacggcccttctcctccgggctgtaattagctgagcaag

aggtaagggtttaagggatggttggttggtggggtattaatgtttaattacctggagcacctgcctgaaatcactttttttcaggtt

gg*Accggtcgccacc*ATGgtgagcaagggcgaggagctgttcaccggggtggtgcccatcctggtcgagctggacg

gcgacgtaaacggccacaagttcagcgtgtccggcgagggcgagggcgatgccacctacggcaagctgaccctgaa

gttcatctgcaccaccggcaagctgcccgtgccctggcccaccctcgtgaccaccctgacctacggcgtgcagtgcttcag

ccgctaccccgaccacatgaagcagcacgacttcttcaagtccgccatgcccgaaggctacgtccagGTAGCCGC

CGCTGAGGCCGAGCCTGCACTGGGGCCACCCAGCCAGGCCTGGGGGCAGCCTC

TCCCCAGCCTCCCCGTGCCAAAAATCTTTTCATTAAAGAATGTTTTGGAACTTTACT

CGCTGGCCTGGCCTTTCTTCTCTCTCCTCCCTATACCTTGAACAGGGAACCCAGGT

GTCTGGGTGCCCTACTCTGGTAAGGAAGGGAGTGGGAACTTTCTGATGCCATGGA

ATATTCCTGTGGGAGCAGTGGACAAGGGTCTGGATTTGTCTTCTGGGAAAGGGAG

GGGAGGACAGACGTGGGGCATGCCCGCCCTGCCTCTCTCCCCCATTCTTGTTGCA

TGCATATCCTCTCATTTCCCTCATTTTTCCTGCAAGAATGTTCTCTCTCATTCCTGAC

CGCCCCTCCACTCCAATTAATAGTGCATGCCTGCTGCCCTACAAGCTTGCTCCCGT

TCTCTCTTCTTTTCCTCTTAAGCTCAGAGTAGCTAGAACAGAGTCAGAGTCACTGCT

CTGGTTCTCTGTCCCCAAGTCTTCCTGAGCCTTCTCCCCTTTTATGTCTTCCCTCTC

CTCCTCCGGGCCCCTAGCCTCCCAAACCCCCATTGCCCGCTGGCTCCTTGGGCAC

AGAACCACACCTTCCTGCCTGGCGGCTGGGAGCCTGCAGGAGCCTGGAGCCTGG

TTGGGCCTGAGTGGTCAGTCCCAGACTCGCCGTCCCGCCTGAGCCTTGTCTCCCT

TCCCAGGAGCGCACCATCTTCTTCAAGGACGACGGCAACTACAAGACCCGCGCCG

AGGTGAAGTTCGAGGGCGACACCCTGGTGAACCGCATCGAGCTGAAGGGCATCG

ACTTCAAGGAGGACGGCAACATCCTGGGGCACAAGCTGGAGTACAACTACAACAG

CCACAACGTCTATATCATGGCCGACAAGCAGAAGAACGGCATCAAGGTGAACTTC

AAGATCCGCCACAACATCGAGGACGGCAGCGTGCAGCTCGCCGACCACTACCAG

CAGAACACCCCCATCGGCGACGGCCCCGTGCTGCTGCCCGACAACCACTACCTG

AGCACCCAGTCCGCCCTGAGCAAAGACCCCAACGAGAAGCGCGATCACATGGTC

CTGCTGGAGTTCGTGACCGCCGCCGGGATCACTCTCGGCATGGACGAGCTGTACA

AGTAA*CATATGTCT*TGATCATAATCAGCCATACCACATTTGTAGAGGTTTTACTTGC

TTTAAAAAACCTCCCACACCTCCCCCTGAACCTGAAACATAAAATGAATGCAATTGT

TGTTGTTAACTTGTTTATTGCAGCTTATAATGGTTACAAATAAAGCAATAGCATCACA

AATTTCACAAATAAAGCATTTTTTTCACTGCATTCTAGTTGTGGTTTGTCCAAACTCA

TCAATGTATCTTA

Supplementary Table 2: List of Primers Used

Primer Number	Primer Name	Primer Sequence
5' Guide RNA Cloning Primers		
1	LMNA 5' Guide 1 F	AGAGCTCGTTGCGATATTATGAAAAGATTTTTGGCACGGGGAGGCTGGGG
2	LMNA 5' Guide 2 F	AGAGCTCGTTGCGATATTATCAAATCCAGACCCTTGTCCACTGCTCCCAC
3	LMNA 5' Guide 3 F	AGAGCTCGTTGCGATATTATAAAAGAAGAGAGAACGGGAGCAAGCTT
4	LMNA 5' Guide 4 F	AGAGCTCGTTGCGATATTATTTCTAGCTACTCTGAGCTTAAGAGGAAAAG
5	LMNA 5' Guide 5 F	AGAGCTCGTTGCGATATTATTCTGGGACTGACCACTCAGGCCCAACCAGG
6	LMNA 5' Guide 1 R	AAAACTGGACCTTCCACAACCCCCAGCCTCCCCGTGCCAAAAATCTTTTC
7	LMNA 5' Guide 2 R	AAAACTGGACCTTCCACAACGTGGGAGCAGTGGACAAGGGTCTGGATTTG
8	LMNA 5' Guide 3 R	AAAACTGGACCTTCCACAACAAGCTTGCTCCCGTTCTCTCTTCTTTT
9	LMNA 5' Guide 4 R	AAAACTGGACCTTCCACAACCTTTTCCTCTTAAGCTCAGAGTAGCTAGAA
10	LMNA 5' Guide 5 R	AAAACTGGACCTTCCACAACCCTGGTTGGGCCTGAGTGGTCAGTCCCAGA
11	RYR2 5' Guide 1 F	AGAGCTCGTTGCGATATTATTGAGTAATGGATGTGAAGTTCCATCAGTGC

12 RYR2 5' Guide 2 F

AGAGCTCGTTGCGATATTATTAGAGAGTTTGCCTAAAGGGACTGACTTGC

13 RYR2 5' Guide 3 F

AGAGCTCGTTGCGATATTATCGAGCATAGCAGCAAAGCACAACTCTAGCG

14 RYR2 5' Guide 4 F

AGAGCTCGTTGCGATATTATGCTGATACTTTAATGGAGAAGAATATGAGC

15 RYR2 5' Guide 5 F

AGAGCTCGTTGCGATATTATGGCTAACATCACATAATTTCCTGCATCATA

16 RYR2 5' Guide 1 R

AAAACTGGACCTTCCACAACGCACTGATGGAACTTCACATCCATTACTCA

17 RYR2 5' Guide 2 R

AAAACTGGACCTTCCACAACGCAAGTCAGTCCCTTTAGGCAAACTCTCTA

18 RYR2 5' Guide 3 R

AAAACTGGACCTTCCACAACCGCTAGAGTTGTGCTTTGCTGCTATGCTCG

19 RYR2 5' Guide 4 R

AAAACTGGACCTTCCACAACGCTCATATTCTTCTCCATTAAAGTATCAGC

20 RYR2 5' Guide 5 R

AAAACTGGACCTTCCACAACTATGATGCAGGAAATTATGTGATGTTAGCC

3' Guide RNA Cloning Primers

21 LMNA 3' Guide 1 F

AACTGGTCGGGGTTTGAAACGAAAAGATTTTTGGCACGGGGAGGCTGGGG

22 LMNA 3' Guide 2 F

AACTGGTCGGGGTTTGAAACCAAATCCAGACCCTTGTCCACTGCTCCCAC

23 LMNA 3' Guide 3 F

AACTGGTCGGGGTTTGAAACAAAAGAAGAGAGAACGGGAGCAAGCTT

24 LMNA 3' Guide 4 F

AACTGGTCGGGGTTTGAAACTTCTAGCTACTCTGAGCTTAAGAGGAAAAG

25 LMNA 3' Guide 5 F

AACTGGTCGGGGTTTGAAACTCTGGGACTGACCACTCAGGCCCAACCAGG

26 LMNA 3' Guide 1 R

GTTATAATAATGTTCCGCGGCCCCAGCCTCCCCGTGCCAAAAATCTTTTC

27 LMNA 3' Guide 2 R

GTTATAATAATGTTCCGCGGGTGGGAGCAGTGGACAAGGGTCTGGATTTG

28 LMNA 3' Guide 3 R

GTTATAATAATGTTCCGCGGAAGCTTGCTCCCGTTCTCTCTTCTTTT

29 LMNA 3' Guide 4 R

GTTATAATAATGTTCCGCGGCTTTTCCTCTTAAGCTCAGAGTAGCTAGAA

30 LMNA 3' Guide 5 R

GTTATAATAATGTTCCGCGGCCTGGTTGGGCCTGAGTGGTCAGTCCCAGA

31 RYR2 3' Guide 1 F

AACTGGTCGGGGTTTGAAACTGAGTAATGGATGTGAAGTTCCATCAGTGC

32 RYR2 3' Guide 2 F

AACTGGTCGGGGTTTGAAACTAGAGAGTTTGCCTAAAGGGACTGACTTGC

33 RYR2 3' Guide 3 F

AACTGGTCGGGGTTTGAAACCGAGCATAGCAGCAAAGCACAACTCTAGCG

34 RYR2 3' Guide 4 F

AACTGGTCGGGGTTTGAAACGCTGATACTTTAATGGAGAAGAATATGAGC

35 RYR2 3' Guide 5 F

AACTGGTCGGGGTTTGAAACGGCTAACATCACATAATTTCCTGCATCATA

36 RYR2 3' Guide 1 R

GTTATAATAATGTTCCGCGGGCACTGATGGAACTTCACATCCATTACTCA

37 RYR2 3' Guide 2 R

GTTATAATAATGTTCCGCGGGCAAGTCAGTCCCTTTAGGCAAACTCTCTA

38 RYR2 3' Guide 3 R

GTTATAATAATGTTCCGCGGCGCTAGAGTTGTGCTTTGCTGCTATGCTCG

39 RYR2 3' Guide 4 R

GTTATAATAATGTTCCGCGGGCTCATATTCTTCTCCATTAAAGTATCAGC

40 RYR2 3' Guide 5 R

GTTATAATAATGTTCCGCGGTATGATGCAGGAAATTATGTGATGTTAGCC

Targeted Amplicon Sequencing Primers

41 DMD HTS F CAGCCTCGTAGTCCTGCCCAGA

42 DMD HTS R AGGAGAGGACACCGTTGTGCCA

43 LMNA HTS F CTGGTGCGCTCAGTGACTGTGG

44 LMNA HTS R GATGCTGCAGTTCTGGGGGCTC

Sanger Sequencing Primers

45 DMD Sanger F TGCCTCGTCCCCTCAGCTTTCA

46 DMD Sanger R GGTCACCTTCAGCTTGGCGGTC

47 LMNA Sanger F CTGGTGCGCTCAGTGACTGTGG

48 LMNA Sanger R CAGAGGCCTGCGGATCCTTAct

SplitGFP Cloning Primers

49 G Forward aacgggtttgccgccagaacacaggCGGATCCATGgtgagcaagggcgaggagctgt

50 G Reverse TTGAAGAAGATGGTGCGCTCctGCTAGCctggacgtagccttcgggcatggcg

51 FP Forward GAGCGCACCATCTTCTTCAAGGACG

52 FP Reverse

GTGGTATGGCTGATTATGATCAAGACATATGTTACTTGTACAGCTCGTCCATGCCGA

53 3' QC ForwardCAAGGACGACTAGAACTACAAGACCCG

54 3' QC ReverseAAGAAGATGGTGCGCTCc

55 5' QC Forward CGGCGAGGGCtagGGCGATGCCA

56 5' QC Reverse GACACGCTGAACTTGTGGCCGTTTACG

57 RYR2 F atgcccgaaggctacgtccagGTGAGTAAATAATTATATGAGACTTTCTG

58 RYR2 R

TTGAAGAAGATGGTGCGCTCCTAAAATATACAAAAGCATAGAAGTAAATA

59 LMNA F tgcccgaaggctacgtccagGTAGCCGCCGCTGAGGCCGAGCCTGCACTG

60 LMNA R

TTGAAGAAGATGGTGCGCTCCTGGGAAGGGAGACAAGGCTCAGGCGGGAC
