# Supplementary material for: Repurposing CRISPR-Cas13 systems for robust mRNA trans-splicing
Source: Nat Commun. 2024 Mar 14;15:2325. doi: 10.1038/s41467-024-46172-4 (PMC10940283; doi:10.1038/s41467-024-46172-4)
Supplement: Supplementary file 2 — Reporting Summary [file 41467_2024_46172_MOESM2_ESM.pdf]

nature portfolio

Corresponding author(s): Aravind Asokan

Last updated by author(s): 02/13/2024

Reporting Summary

Nature Portfolio wishes to improve the reproducibility of the work that we publish. This form provides structure for consistency and transparency in reporting. For further information on Nature Portfolio policies, see our Editorial Policies and the Editorial Policy Checklist.

Statistics

For all statistical analyses, confirm that the following items are present in the figure legend, table legend, main text, or Methods section.

n/a	Confirmed	
☐	☒	The exact sample size (*n*) for each experimental group/condition, given as a discrete number and unit of measurement
☐	☒	A statement on whether measurements were taken from distinct samples or whether the same sample was measured repeatedly
☐	☒	The statistical test(s) used AND whether they are one- or two-sided<br>*Only common tests should be described solely by name; describe more complex techniques in the Methods section.*
☒	☐	A description of all covariates tested
☒	☐	A description of any assumptions or corrections, such as tests of normality and adjustment for multiple comparisons
☒	☐	A full description of the statistical parameters including central tendency (e.g. means) or other basic estimates (e.g. regression coefficient) AND variation (e.g. standard deviation) or associated estimates of uncertainty (e.g. confidence intervals)
☐	☒	For null hypothesis testing, the test statistic (e.g. *F*, *t*, *r*) with confidence intervals, effect sizes, degrees of freedom and *P* value noted<br>*Give P values as exact values whenever suitable.*
☒	☐	For Bayesian analysis, information on the choice of priors and Markov chain Monte Carlo settings
☒	☐	For hierarchical and complex designs, identification of the appropriate level for tests and full reporting of outcomes
☒	☐	Estimates of effect sizes (e.g. Cohen's *d*, Pearson's *r*), indicating how they were calculated

Our web collection on statistics for biologists contains articles on many of the points above.

Software and code

Policy information about availability of computer code

Data collection	All next generation sequencing data was collected using an Illumina HiSeq machine as FASTQ files. Flow cytometry was performed on a sony MA900. Immunofluorescence images were collected on either a Zeiss 780 or Leica sp8 confocal microscope.
Data analysis	Differential gene expression was completed using a previously published pipeline available here on github: https://aaronmitchd.github.io/RNA_Seq/index.html. All off-target analysis was completed using Star Aligner package Arriba version 2.4.0. Statistical analysis was performed with Graphpad Prism version 9.5.0. On target editing analysis outcomes were analyzed using CRISPResso2.0. Flow cytometry data was analyzed in FlowJo version 10.8.1. Immuno-fluorescent images were analyzed in ImageJ version 2.1.0. A custom python script was made to analyze the barcode library and is available at https://github.com/dnf97/CRAFT.

For manuscripts utilizing custom algorithms or software that are central to the research but not yet described in published literature, software must be made available to editors and reviewers. We strongly encourage code deposition in a community repository (e.g. GitHub). See the Nature Portfolio guidelines for submitting code & software for further information.

Data

Policy information about availability of data

All manuscripts must include a data availability statement. This statement should provide the following information, where applicable:

- Accession codes, unique identifiers, or web links for publicly available datasets
- A description of any restrictions on data availability
- For clinical datasets or third party data, please ensure that the statement adheres to our policy

High-throughput sequencing data have been deposited in the NCBI Sequence Read Archive database under the accession code PRJNA1076184. All other data associated with this study are present in the paper, supplementary materials, or source data. The data that support the findings of this study are available from the corresponding author upon reasonable request. Correspondence and requests for materials should be addressed to A.A. at aravind.asokan@duke.edu.

Human research participants

Policy information about studies involving human research participants and Sex and Gender in Research.

Reporting on sex and gender	N/A
Population characteristics	N/A
Recruitment	N/A
Ethics oversight	N/A

Note that full information on the approval of the study protocol must also be provided in the manuscript.

Field-specific reporting

Please select the one below that is the best fit for your research. If you are not sure, read the appropriate sections before making your selection.

- [x] Life sciences
- [ ] Behavioural & social sciences
- [ ] Ecological, evolutionary & environmental sciences

For a reference copy of the document with all sections, see nature.com/documents/nr-reporting-summary-flat.pdf

Life sciences study design

All studies must disclose on these points even when the disclosure is negative.

Sample size	For in vitro experiments n = 3 biological replicates. This sample size was determined based on literature precedence for RNA editing experiments. For the mouse experiment a power analysis was performed to determine n = 4 would be sufficient for determining the results of the experiment, and is consistent with literature on the mouse model used.
Data exclusions	No data was excluded
Replication	All in vitro experiments were performed at least two times with a sample size of n = 3 and are consistent with the results shown in the manuscript. Mouse experiments were performed only once. All attempts at replication were successful
Randomization	Randomization was not performed for in vitro experiments as it is not relevant due to the fact that clonal cell lines are used. Randomization was not performed for this experiment as inbred mouse lines were used for this experiment.
Blinding	Blinding was performed for immunofluoresence imaging analysis. Images were stripped of identifying information and quantified in ImajeJ with a uniform automated protocol that was constant for all images collected.

Reporting for specific materials, systems and methods

We require information from authors about some types of materials, experimental systems and methods used in many studies. Here, indicate whether each material, system or method listed is relevant to your study. If you are not sure if a list item applies to your research, read the appropriate section before selecting a response.

Materials & experimental systems

n/a	Involved in the study	
☐	☒	Antibodies
☐	☒	Eukaryotic cell lines
☒	☐	Palaeontology and archaeology
☒	☐	Animals and other organisms
☒	☐	Clinical data
☒	☐	Dual use research of concern

Methods

n/a	Involved in the study	
☒	☐	ChIP-seq
☐	☒	Flow cytometry
☒	☐	MRI-based neuroimaging

Antibodies

Antibodies used	Primary antibodies: Lamin A antibody (1:500; Abcam, ab40567), rabbit anti-Flag (1:500; Cell Signaling, #14793), Anti-HA.c5 antibody (1:500; MA5-27543), anti-beta actin antibody (MA1-91399), rabbit anti-dystrophin (1:100; abcam 275391) and rat anti-laminin (1:400; Sigma L0663).<br><br>Secondary antibodies: AlexaFluor488 goat anti-rabbit (1:500; Thermo Fisher Scientific, A-11008), AlexaFluor594 goat anti-mouse (1:500; Thermo Fisher Scientific, A-11005), and anti-rabbit IgG AlexaFluor 647 (1:400; Invitrogen) and goat anti-rat IgG AlexaFluor 594 (1:500; Invitrogen).
Validation	Lamin A antibody (Abcam, ab40567), was confirmed by staining fibroblasts of healthy donors and HGPS patients in Koblan, L. W. et al. In vivo base editing rescues Hutchinson–Gilford progeria syndrome in mice. Nature 589, 608–614 (2021).<br><br>Rabbit anti-dystrophin (1:100; abcam 275391) was validated by western blot and immunohistochemistry on manufacturer website.<br><br>rat anti-laminin (1:400; Sigma L0663) was validated immunohistochemistry staining of healthy donor and DMD patient samples Tatyana, A. et al. Automated immunofluorescence analysis for sensitive and precise dystrophin quantification in muscle biopsies.<br><br>Rabbit anti-Flag (Cell Signaling, #14793) and Anti-HA.c5 antibody (MA5-27543) were validated by western blot in Elmore, Z. C. et al. “The membrane associated accessory protein is an adeno-associated viral egress factor.” Nature communications vol. 12,1 6239. 29 Oct. 2021, doi:10.1038/s41467-021-26485-4

Eukaryotic cell lines

Policy information about cell lines and Sex and Gender in Research

Cell line source(s)	HEK 293, Hep G2, A549, and HeLa cells were acquired from ATCC. HPGS patient-derived cell line was obtained from the Coriell Institute cell line (AG06917).
Authentication	HEK 293, Hep G2, A549, and HeLa cells were authenticated by supplier using STR analysis.
Mycoplasma contamination	Cell lines tested negative for mycoplasma.
Commonly misidentified lines (See ICLAC register)	No commonly misidentified cell lines were used in this study

Flow Cytometry

Plots

Confirm that:

☒ The axis labels state the marker and fluorochrome used (e.g. CD4-FITC).

☒ The axis scales are clearly visible. Include numbers along axes only for bottom left plot of group (a 'group' is an analysis of identical markers).

☒ All plots are contour plots with outliers or pseudocolor plots.

☒ A numerical value for number of cells or percentage (with statistics) is provided.

Methodology

Sample preparation	cells were harvested 48 hours post transfection with 100ul trypsin, and diluted in 500ul PBS with 10%FBS. Cells were then passed through a 100um filter.
Instrument	Sony MA900

Software	FlowJo version 10.8.1
Cell population abundance	10,000 events were collected for each sample, each gate indicates the percent events.
Gating strategy	FSC/SSC was used for identifying cell populations, this was then passed to FSC-A/FSC-H for doublet discrimination, this gate was then passed FSC-A/FITC for identification of EGFP positive cells.

☒ Tick this box to confirm that a figure exemplifying the gating strategy is provided in the Supplementary Information.
